# Mosquito survey in Mauritania: Detection of Rift Valley fever virus and dengue virus and the determination of feeding patterns

**DOI:** 10.1371/journal.pntd.0010203

**Published:** 2022-04-15

**Authors:** Franziska Stoek, Yahya Barry, Aliou Ba, Ansgar Schulz, Melanie Rissmann, Claudia Wylezich, Balal Sadeghi, Abdellahi Diambar Beyit, Albert Eisenbarth, Fatimetou Bounene N’diaye, Mohamed Lemine Haki, Baba Abdellahi Doumbia, Mohamed Baba Gueya, Mohamed Yahya Bah, Martin Eiden, Martin H. Groschup

**Affiliations:** 1 Institute of Novel and Emerging Infectious Diseases, Friedrich-Loeffler-Institut, Greifswald-Insel Riems, Germany; 2 Office National de Recherche et de Développement de l’Elevage (ONARDEL), Nouakchott, Mauritania; 3 Institute of Diagnostic Virology, Friedrich-Loeffler-Institut, Greifswald-Insel Riems, Germany; 4 Ministère du Développement Rural, Nouakchott, Mauritania; Oregon State University College of Veterinary Medicine, UNITED STATES

## Abstract

In Mauritania, several mosquito-borne viruses have been reported that can cause devastating diseases in animals and humans. However, monitoring data on their occurrence and local distribution are limited. Rift Valley fever virus (RVFV) is an arthropod-borne virus that causes major outbreaks throughout the African continent and the Arabian Peninsula. The first Rift Valley fever (RVF) epidemic in Mauritania occurred in 1987 and since then the country has been affected by recurrent outbreaks of the disease. To gain information on the occurrence of RVFV as well as other mosquito-borne viruses and their vectors in Mauritania, we collected and examined 4,950 mosquitoes, belonging to four genera and 14 species. The mosquitoes were captured during 2018 in the capital Nouakchott and in southern parts of Mauritania. Evidence of RVFV was found in a mosquito pool of female *Anopheles pharoensis* mosquitoes collected in December on a farm near the Senegal River. At that time, 37.5% of 16 tested Montbéliarde cattle on the farm showed RVFV-specific IgM antibodies. Additionally, we detected IgM antibodies in 10.7% of 28 indigenous cattle that had been sampled on the same farm one month earlier. To obtain information on potential RVFV reservoir hosts, blood meals of captured engorged mosquitoes were analyzed. The mosquitoes mainly fed on humans (urban areas) and cattle (rural areas), but also on small ruminants, donkeys, cats, dogs and straw-colored fruit bats. Results of this study demonstrate the circulation of RVFV in Mauritania and thus the need for further research to investigate the distribution of the virus and its vectors. Furthermore, factors that may contribute to its maintenance should be analyzed more closely. In addition, two mosquito pools containing *Aedes aegypti* and *Culex quinquefasciatus* mosquitoes showed evidence of dengue virus (DENV) 2 circulation in the city of Rosso. Further studies are therefore needed to also examine DENV circulation in Mauritania.

## Author summary

In Mauritania, various mosquito-borne viruses responsible for devastating diseases in livestock and/or humans occur, threatening both human and animal health. One of these viruses is the Rift Valley fever phlebovirus (RVFV), which is widespread throughout the African continent and the Arabian Peninsula. The first outbreak in Mauritania occurred in 1987, and epidemics have recurred since then. However, little is known about the maintenance of RVFV during inter-epidemic periods, in the absence of reported outbreaks, and although there is evidence of an active circulation of the virus during these times, only limited data are available on the abundance of RVFV and its vectors in Mauritania. Therefore, this study aimed to collect and examine mosquitoes in different areas where outbreaks have already occurred. Additionally, blood of collected blood-fed mosquitoes was analyzed to identify feeding patterns and thus wildlife potentially acting as RVFV reservoir hosts. Furthermore, the collected mosquitoes were analyzed for the presence of other neglected viruses in order to participate in an elucidation of the circulation of different viruses within the mosquito population in Mauritania and to be able to better prevent disease outbreaks.

## I. Introduction

*Rift Valley fever phlebovirus* is an arthropod-borne virus (arbovirus) of the order *Bunyavirales*, family *Phenuiviridae*, genus *Phlebovirus*, causing recurrent epidemics throughout Africa and the Arabian Peninsula [1]. It is an enveloped RNA virus with a tripartite genome (S, M and L segments) that encodes a nucleoprotein (NP), two nonstructural proteins (NSs, NSm), two glycoproteins (Gc, Gn) and an RNA-dependent RNA polymerase [2]. First described in Kenya in 1931 [3], the virus induces severe disease in ruminants and humans with great impact on health and economy systems. In ruminants, the so-called “abortion-storms” with newborn fatality rates of up to 100% are characteristic of Rift Valley fever virus (RVFV) infection. Humans mostly develop only flu-like febrile illness with mild symptoms, but severe manifestations with neurological disorders, blindness or fatal hemorrhagic fever can occur [4].

The RVFV transmission cycle is divided into an enzootic (inter-epidemic) and an epidemic cycle. During the enzootic cycle, the arbovirus is suspected of being maintained by vertical transmission within the mosquito population (*Aedes* spp.) and by sporadic infections of susceptible animals [1]. A wide range of vertebrates other than ruminants and humans are known to be susceptible to the virus, and it is assumed that wildlife plays a role in RVFV maintenance during inter-epidemic periods. However, a specific reservoir host has not been identified to date [5]. Blood meal analyses of RVFV vectors represent a useful molecular tool to identify animals that may be involved in the maintenance of the virus, as feeding hosts can contribute to sustain the RVFV transmission cycle. The epidemic cycle generally occurs when climatic changes, such as heavy rainfall or inundations, favor an increase in the mosquito population, resulting in an enhanced likelihood of RVFV transmission to susceptible hosts. These infected hosts then transmit the virus to other vertebrates and mosquitoes, which in turn can spread the virus [1]. In the mid-1950s, the virus was first isolated from mosquitoes [6] and since then, RVFV isolation from over 40 different mosquito species has been described [7,8], indicating the potential involvement of these mosquitoes in the RVFV ecology.

In Mauritania, the first Rift Valley fever (RVF) epidemic occurred in 1987 [9], but virological and serological findings point to an introduction of the virus into West Africa before this outbreak [10]. Thereafter, the country has been affected by recurrent RVF outbreaks threatening the population and its essential livestock [11,12]. The occurrence of more than ten of the mosquito species believed to play a role in RVFV ecology has been described in Mauritania over the past 20 years [8,12]. During outbreaks in 1998 and 2003, *Culex poicilipes* and *Culex antennatus* mosquitoes were found to be infected with RVFV [13,14]. Additionally, the virus was isolated from *Cx*. *poicilipes* during an inter-epidemic period [15]. Together with the evidence of RVFV infection found in cattle, this demonstrates an active circulation of the virus during the absence of reported epidemics [16]. However, only scarce information on the abundance of RVFV-transmitting mosquitoes within Mauritania exist [12], and data on RVFV maintenance during the enzootic cycle are limited [1].

In addition to RVFV, various other mosquito-borne viruses such as Ngari virus (*Orthobunyavirus*), Wesselsbron virus (*Flavivirus*), yellow fever virus (*Flavivirus*) or dengue virus (*Flavivirus*), responsible for devastating diseases in livestock and/or humans, are occurring in Mauritania [15,17,18]. With estimated 390 million infections per year, dengue fever is the most frequent arboviral disease in humans worldwide. Although the virus is endemic in most tropical and sub-tropical regions of the world, its importance in Mauritania and other African countries may be largely underestimated [19,20]. Serotypes of dengue virus (DENV) have been isolated from humans, mosquitoes and monkeys in Mauritania’s neighboring countries Senegal and Mali [21,22,23,24]. However, dengue fever has only recently been reported in Mauritania [12,25] and there is a lack of available data on the circulation of the virus in the country. Moreover, many other arboviruses are probably neglected due to the symptomatic similarities of the diseases and poor surveillance [11,20].

In this study, sampling and analyses of mosquitoes were performed to obtain information on the occurrence and distribution of RVFV and its vectors in Mauritania. To elucidate factors that may favor the maintenance of RVFV during inter-epidemic periods, mosquitoes were sampled in 2018 mainly in the absence of RVF, but also during an epizootic on a farm in southern Mauritania. Captured engorged mosquitoes were additionally examined by blood meal analysis to identify preferred hosts of the mosquitoes and thus a potential RVFV reservoir. Furthermore, we aimed to gain insights into the distribution and circulation of other important arboviruses within mosquito populations in Mauritania. Therefore, mosquito samples were also analyzed for evidence of flavi- and orthobunyaviruses.

## II. Methods

### II.I. Ethics statement

The collection of blood samples was carried out by the Mauritanian State Veterinary Laboratory, the Office National de Recherches et de Développement de l’Elevage (ONARDEL), in compliance with all relevant national and international regulations and fundamental ethical principles for diagnostic purposes, within the framework of a governmental animal health surveillance program (Ministère de Développement Rural).

### II.II. Collection sites

**Fig 1:**
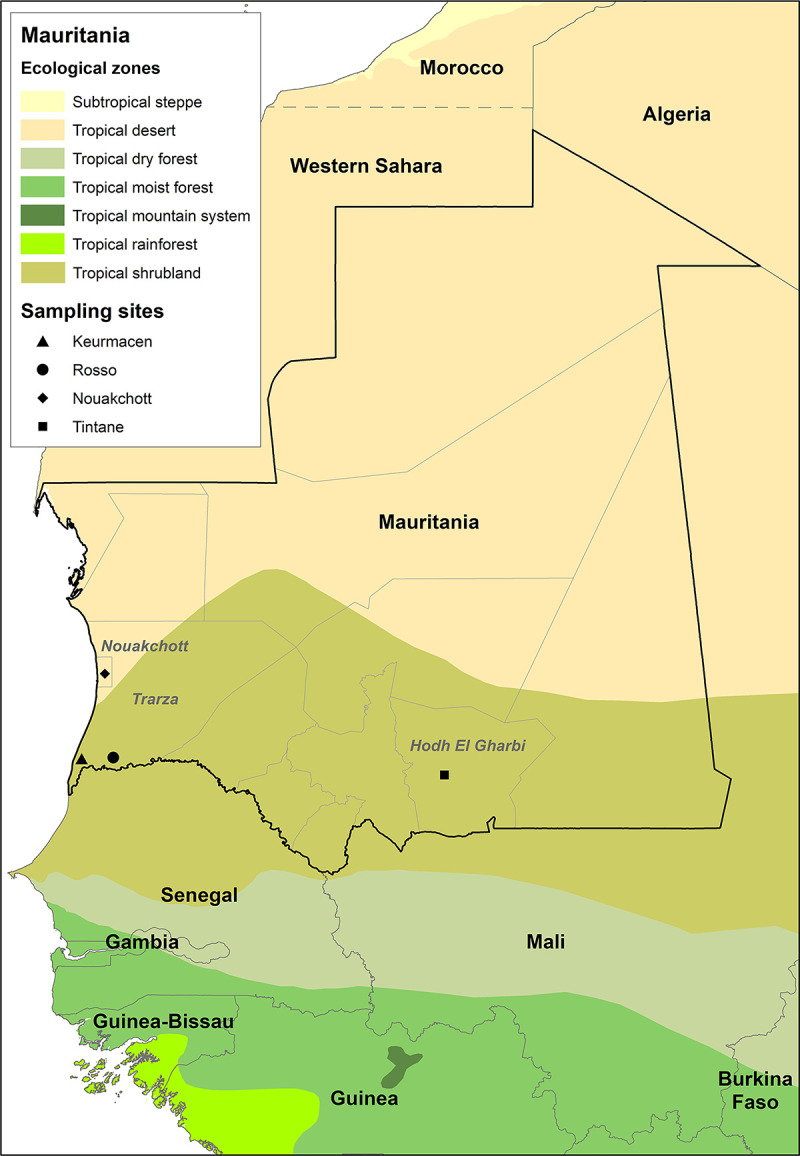
Selected sampling sites. Several locations were sampled in Nouakchott and Rosso, which are not plotted individually on the map due to scale. The map was created with GADM data 3.6 (https://gadm.org/download_world.html) and the FAO Global Ecological Zones (GEZ) (https://data.apps.fao.org/map/catalog/srv/ger/catalog.search#/metadata/2fb209d0-fd34-4e5e-a3d8-a13c241eb61b). Direct link to the base layer: https://biogeo.ucdavis.edu/data/gadm3.6/gadm36_gpkg.zip.

In the course of 2018, mosquitoes were collected in Mauritania. The largest part of the West African country is characterized by the arid desert landscape of the Sahara. Only in the southern regions of Mauritania, where the Senegal River marks the border with Senegal, a more humid climate (semi-arid climate of the Sahel) prevails [12], favoring the occurrence of high mosquito densities. Therefore, in this study mosquitoes were collected in three southern regions of Mauritania: in Nouakchott, the capital and its surroundings (four locations: Socogim Le Ksar; Botanical Garden Sebkha; Basra Sebkha; Arafat); in Trarza (three locations: Rosso, in and outside the city; Keurmacen); and in Hodh El Gharbi (one location: Tintane) (**Fig 1**). In December 2018, during the rainy season, symptoms indicative for RVF were observed on a cattle farm in Rosso, which is why the farm was selected as sampling site. Altogether, mosquito traps were set at eight different dates at eight different locations. In Nouakchott, the traps were set twice at two of the locations (Socogim Le Ksar and the Botanical Garden in Sebkha). Thus, samples were taken at a total of ten different sampling sites, differing in date and/or location.

### II.III. Mosquito collection and species identification

Mosquitoes were collected with Heavy Duty EVS (Encephalitis Vector Survey) CO_2_ Mosquito Traps (BioQuip Products Inc., Rancho Dominguez, CA, USA). Carbon dioxide (CO_2_) filled bottles were placed next to traps and CO_2_ was directed to the traps with hoses to attract mosquitoes. The traps were set up at dusk in sheltered places and run over night. At dawn, bags with captured mosquitoes were gathered and subsequently transferred to -20°C. Frozen mosquitoes were placed on chill tables and morphologically identified to species (or genus) levels using taxonomic keys by *Peter F*. *Mattingly* and *Assane G*. *Fall* [26,27]. Male mosquitoes were identified to genus level only. After identification, mosquitoes were stored at -80°C in pools of up to ten mosquitoes of the same species/genus, sex and sampling site. Blood-fed mosquitoes (with a visible blood meal in the abdomen) were separated and stored individually. For pathogen examination, cooled samples were shipped to the Friedrich-Loeffler-Institut (FLI). At FLI, samples were stored at -80°C until further processing.

### II.IV. Serum collection

As mentioned above (II.II.), in December 2018, Montbéliarde cattle on a farm situated 15 km south of the town of Rosso (**Fig 1**), near the Senegal River, showed clinical symptoms indicative of RVFV infection. To confirm the clinically observed RVF disease, sera were taken from 16 individuals of the herd. In addition, in order to gain further insight into the onset of this epizootic, 28 sera from indigenous cattle on the same farm that had been collected during a routine medical examination one month earlier were analyzed. For protection of cattle and humans, animals were carefully captured and restrained during blood collection.

### II.V. RNA/DNA extraction

Three 3-mm steel beads and 500 μl (mosquito pools) or 300 μl (individual mosquitoes) Minimal Essential Medium (MEM) with penicillin, streptomycin, amphotericin and gentamicin were added to the samples. Mosquitoes were homogenized using a TissueLyser II (Qiagen, Hilden, Germany) at 30 Hz for 2 minutes, and then centrifuged at 4°C, 13,000 rpm for 3 minutes. Supernatants were used for RNA/DNA extraction with the NucleoMag VET kit (MACHEREY-NAGEL GmbH & Co. KG, Düren, Germany) according to the manufacturer’s recommendations. Residues of the supernatants were stored at -80°C. Before conducting the extraction, an MS2 bacteriophage was added to each sample as an internal extraction control [28].

### II.VI. Pathogen screening

RNA/DNA of all obtained mosquito and serum samples were tested for the presence of RVFV-specific RNA as well as for RNA from flaviviruses and orthobunyaviruses.

#### II.VI.I. Rift Valley fever phlebovirus detection

A quantitative real-time PCR (qRT-PCR) [29] using the QuantiTect Probe RT-PCR Kit (Qiagen, Hilden, Germany) was performed to verify the presence of RVFV-specific RNA. To quantify the present viral RNA, a synthetic RNA calibrator was used [30]. Samples that contained more than one copy/μl (5 copies/reaction) of RVFV-specific RNA were considered as positive [29].

#### II.VI.II. Pan-*Flavivirus* detection

A one-step SYBR Green-based qRT-PCR with melting curve analysis [31] was conducted using the QuantiTect SYBR Green RT-PCR Kit (QIAGEN, Hilden, Germany) to detect RNA from viruses of the genus *Flaviviru*s. The non-purified PCR products of samples showing a specific melting curve (positive control: Usutu virus-derived RNA) were sent to Eurofins Genomics (Eurofins Genomics GmbH, Ebersberg, Germany) for DNA sequencing by the Sanger method using the amplification primers. Obtained sequences were analyzed with BLAST search (https://blast.ncbi.nlm.nih.gov/Blast.cgi, NCBI, Bethesda, USA) to identify virus strains.

#### II.VI.III. Pan-Orthobunyavirus detection

Another one-step SYBR Green-based qRT-PCR with melting curve analysis [17] using the QuantiTect SYBR Green RT-PCR Kit (QIAGEN, Hilden, Germany) was performed to detect RNA from viruses of the genus *Orthobunyavirus* (positive control: Bunyamwera virus-derived RNA). Following steps were conducted as described before (II.VI.II.).

### II.VII. Blood meal analysis

Extracted RNA/DNA from blood-fed mosquitoes was additionally used for blood meal analysis to infer host preferences of mosquito species and thus to determine possible reservoir hosts of RVFV.

A PCR specific for mitochondrial cytochrome b of game species [32] was performed using the QuantiTect Multiplex PCR NoROX Kit (QIAGEN, Hilden, Germany). PCR products were verified by gel electrophoresis in a 1.5% agarose gel. A 100 bp DNA ladder (peqGOLD, VWR International (Part of Avantor), Pennsylvania, USA) was used to identify specific PCR products (464 bp).

Additionally, a PCR specific for mitochondrial 16S ribosomal RNA of vertebrates was carried out using previously published primers [33]. The PCR protocol was modified from *Schlegel* et al. [34]. A reaction volume of 25 μl containing 3 μl of template DNA, 10 pmol of each primer, 25 mM MgCl_2_, 10 mM dNTP Mix, 5× GoTaq buffer and 5 U GoTaq G2 Flexi DNA Polymerase (Promega Corporation, Fitchburg, WI, USA) was used. Verification of PCR products was performed by gel electrophoresis in a 1.5% agarose gel. To identify specific PCR products (244 bp), a 200 bp DNA ladder (Jena Bioscience GmbH, Jena, Germany) was used.

Gels were stained with ethidium bromide for evaluation. PCR products of samples showing a specific band in the gel were sent for sequencing and analyzed as described before (II.VI.II.).

### II.VIII. IgM Capture ELISA

Heat-inactivated cattle sera were tested for the presence of IgM antibodies against RVFV NP with the ID Screen Rift Valley Fever IgM Capture ELISA for ruminants (IDvet, Grabels, France). The ELISA was conducted according to the manufacturer’s instructions.

### II.IX. Virus isolation

For qRT-PCR-positive mosquito samples, propagation of the virus on cells was attempted. To prevent secondary cell infection, penicillin, streptomycin, amphotericin and gentamicin were added to culture medium (MEM).

#### II.IX.I. Mosquitoes

100 μl of the supernatants (II.V.) of mosquito samples that had tested positive in qRT-PCR were diluted in 900 μl MEM and subsequently filtered (Millex-GP, 0.22 μm; Merck KGaA, Darmstadt, Germany). 400 μl each of the filtered samples were added to a well of a 6-well plate with 90% confluent monolayers of Vero 76 cells (African green monkey kidney cells, Collection of Cell Lines in Veterinary Medicine, Friedrich-Loeffler-Institut, Germany) and to a well of a 6-well plate with 90% confluent monolayers of C6/36 cells (*Aedes albopictus* larval cells, Collection of Cell Lines in Veterinary Medicine, Friedrich-Loeffler-Institut, Germany). Cells were incubated at 37°C (Vero 76) or 28°C (C6/36) and 5% CO_2_ respectively, and checked daily for the presence of a cytopathogenic effect (CPE). Cell culture supernatants were harvested and tested in each of the two qRT-PCRs after a seven-day incubation or until CPE was detected. 400 μl of these cell culture supernatants were again passaged on 90% confluent monolayers of both Vero 76 and C6/36 cells. After incubation for one week, cell culture supernatants were tested in qRT-PCR for the presence of viral RNA (as described before).

#### II.IX.II. Serum samples

50 μl of the serum samples that showed evidence of IgM antibodies against RVFV were diluted in 150 μl MEM and added each to a well of a 12-well plate with 90% confluent monolayers of Vero 76 cells. Cells were kept at 37°C, 5% CO_2_ and checked daily for the presence of CPE. After a maximum of seven days, cell culture supernatants were harvested and tested in the qRT-PCR for the presence of RVFV-specific RNA.

### II.X. Indirect RVFV immunofluorescence assay (IIFA)

Vero 76 cells that have been exposed to RVFV qRT-PCR-positive mosquito supernatants or IgM-positive sera for one week (II.IX.) were fixed with 4% paraformaldehyde. Subsequently, an IIFA to verify RVFV infection of these cells was performed as previously described [35]. A monoclonal antibody against RVFV NP was used as first antibody. Fixed cells infected with RVFV 35/74 strain (accession number: JF784386-88) served as positive control.

### II.XI. High-throughput sequencing

qRT-PCR-positive samples were used for high-throughput sequencing (HTS) to obtain more sequence information. A combination of enrichment of viral nucleic acids with RNA baits and following HTS was applied. For this purpose, RNA extracts were subjected to library preparation as detailed described [36]. Virus nucleic acids were subsequently enriched using myBaits (Arbor Biosciences, Ann Arbor, MI, USA) with the VirBaits panel according to *Wylezich* et al. [37] with a hybridization time of 24 hours at 65°C. The bait panel includes 539 baits specific for RVFV. An extended panel (VirBaits 2.0) was used for the dengue virus containing samples that includes 2804 baits specific for dengue virus. The Genome Sequencer software suite (versions 2.6; Roche) was applied to execute reference mapping analyses. Genome sequences of RVFV (KX944814, KX944837, KX944860) and DENV (MT261970, MT981085) were used as reference. Obtained contigs were analyzed with BLAST search (https://blast.ncbi.nlm.nih.gov/Blast.cgi) to characterize virus sequences.

### II.XII. Phylogenetic analysis

CLUSTAL W was used to align the sequences as implemented in MEGA v.11 software [38]. The best fitting nucleotide substitution model was identified as GTR. Maximum Likelihood (ML) trees were reconstructed using MEGA v.11 software with bootstrap support of 1000 replicates. Finalized trees were visualized using in the FigTree v.1.4.3 program [39].

## III. Results

### III.I. Mosquitoes

#### III.I.I. Collection and species identification

A total of 4,950 mosquitoes of four genera and 14 species were collected and identified in this study (**Table 1**). These included 571 male and 4,379 female mosquitoes, with 258 of the latter having taken a blood meal. Unengorged mosquitoes were grouped into 521 pools, of which 456 pools contained females and 65 pools contained males. Since engorged mosquitoes were analyzed individually, a total of 779 mosquito samples were examined. Comparing the sampling regions, 59.6% of specimens were collected in Nouakchott and its surroundings, while 36.9% were collected in Trarza and 3.5% in Hodh El Gharbi. Looking at the sampling over the year, most mosquitoes were captured in July and December (**S1 Fig**).

**Table 1:** Total number of collected mosquitoes.

Genus	Species	no.	(UG/BF)	%
*Culex*	*antennatus* ^◊^	**9**	(6/3)	**0.18**
	*decens*	**38**	(33/5)	**0.77**
	*duttoni*	**8**	(8/0)	**0.16**
	*neavei* ^◊^	**140**	(140/0)	**2.83**
	*poicilipes* ^◊^	**696**	(695/1)	**14.06**
	*quinquefasciatus* ^◊^	**1882**	(1759/123)	**38.02**
	*tritaeniorhynchus* ^◊^	**589**	(584/5)	**11.90**
	*univittatus* ^◊^	**204**	(168/36)	**4.12**
	NI (f)	**21**	(21/0)	**0.42**
	NI (m)	**551**	(551/-)	**11.13**
*Aedes*	*vexans* ^◊^	**45**	(29/16)	**0.91**
	*aegypti* ^◊^	**13**	(10/3)	**0.26**
	NI (f)	**5**	(5/0)	**0.10**
	NI (m)	**1**	(1/-)	**0.02**
*Anopheles*	*pharoensis* ^◊^	**531**	(472/59)	**10.73**
	*gambiae* ^◊^	**11**	(8/3)	**0.22**
	*ziemanni*	**11**	(11/0)	**0.22**
	NI (f)	**27**	(26/1)	**0.55**
	NI (m)	**19**	(19/-)	**0.38**
*Mansonia*	*uniformis* ^◊^	**149**	(146/3)	**3.01**
Total		**4950**	(4692/258)	**100.00**

NI = (species) not identified

(m) = male mosquitoes

(f) = female mosquitoes

UG = unengorged mosquitoes (without visible blood meal)

BF = blood-fed mosquitoes

^◊^ = natural infection with RVFV has been described [8]

The majority of mosquitoes belonged to the genus *Culex* spp. (4,138 individuals), followed by the genera *Anopheles* spp. (599 individuals) and *Mansonia* spp. (149 individuals). Fewest mosquitoes were collected within the genus *Aedes* spp. (64 individuals). The most frequently occurring mosquito species was *Culex quinquefasciatus* (38%), followed by *Culex poicilipes* (15%) and *Culex tritaeniorhynchus* (12%). Within the other genera, *Anopheles pharoensis (*11%) represented the most abundant species (**Table 1**). Comparing the sampling regions, *Cx*. *quinquefasciatus* represented the most common species in Nouakchott (**Table 2**) and Hodh El Gharbi (**Table 3**), while in Trarza the majority of mosquitoes consisted of *Cx*. *poicilipes* and *An*. *pharoensis* (**Table** **4**). Collection at most sampling sites was performed only once during the study. However, in Nouakchott two locations (Socogim Le Ksar and the Botanical Garden in Sebkha) were sampled twice on different dates (**Table 2**). For both locations, the most frequently collected species remained the same at both times (*Cx*. *quinquefasciatus*), but the abundance of other species changed depending on the date of sampling.

**Table 2:** Number of collected mosquitoes in Nouakchott and its surroundings.

Mosquito genus	Mosquito species	Socogim Le Ksar		Socogim Le Ksar		Botanical Garden Sebkha		Botanical Garden Sebkha		Basra Sebkha		Arafat		Total		
		01.02.2018		08.07.2018		06.05.2018		04.07.18		24.11.2018		02.12.2018				
		no.	(UG/BF)	no.	(UG/BF)	no.	(UG/BF)	no.	(UG/BF)	no.	(UG/BF)	no.	(UG/BF)	no.	(UG/BF)	%
*Culex*	*antennatus*	**0**	(0/0)	**0**	(0/0)	**0**	(0/0)	**2**	(2/0)	**0**	(0/0)	**5**	(2/3)	**7**	(4/3)	**0.24**
	*decens*	**0**	(0/0)	**5**	(5/0)	**0**	(0/0)	**22**	(22/0)	**0**	(0/0)	**0**	(0/0)	**27**	(27/0)	**0.91**
	*neavei*	**40**	(40/0)	**87**	(87/0)	**1**	(1/0)	**2**	(2/0)	**4**	(4/0)	**0**	(0/0)	**134**	(134/0)	**4.54**
	*poicilipes*	**0**	(0/0)	**0**	(0/0)	**0**	(0/0)	**123**	(123/0)	**0**	(0/0)	**0**	(0/0)	**123**	(123/0)	**4.17**
	*quinquefasciatus*	**137**	(137/0)	**158**	(150/8)	**425**	(424/1)	**621**	(567/54)	**2**	(2/0)	**34**	(9/25)	**1377**	(1289/88)	**46.65**
	*tritaeniorhynchus*	**86**	(86/0)	**4**	(4/0)	**70**	(70/0)	**361**	(359/2)	**0**	(0/0)	**0**	(0/0)	**521**	(519/2)	**17.65**
	*univittatus*	**0**	(0/0)	**82**	(61/21)	**4**	(4/0)	**89**	(88/1)	**0**	(0/0)	**18**	(4/14)	**193**	(157/36)	**6.54**
	NI (f)	**11**	(11/0)	**0**	(0/0)	**2**	(2/0)	**4**	(4/0)	**0**	(0/0)	**0**	(0/0)	**17**	(17/0)	**0.58**
	NI (m)	**2**	(2/-)	**26**	(26/-)	**53**	53/-)	**379**	(379/-)	**14**	(14/-)	**10**	(10/-)	**484**	(484/-)	**16.40**
*Aedes*	*vexans*	**7**	(7/0)	**21**	21/0)	**0**	(0/0)	**6**	(1/5)	**0**	(0/0)	**0**	(0/0)	**34**	(29/5)	**1.15**
	*aegypti*	**0**	(0/0)	**0**	(0/0)	**0**	(0/0)	**2**	(2/0)	**0**	(0/0)	**0**	(0/0)	**2**	(2/0)	**0.07**
*Anopheles*	NI (f)	**0**	(0/0)	**0**	(0/0)	**3**	(3/0)	**20**	(20/0)	**0**	(0/0)	**0**	(0/0)	**23**	(23/0)	**0.78**
	NI (m)	**0**	(0/-)	**0**	(0/-)	**0**	(0/-)	**10**	(10/-)	**0**	(0/-)	**0**	(0/-)	**10**	(10/-)	**0.34**
Total		**283**	(283/0)	**383**	(354/29)	**558**	(557/1)	**1641**	(1579/62)	**20**	(20/0)	**67**	(25/42)	**2952**	(2818/134)	**100.00**

NI = (species) not identified

(m) = male mosquitoes

(f) = female mosquitoes

UG = unengorged mosquitoes (without visible blood meal)

BF = blood-fed mosquitoes

**Table 3:** Number of collected mosquitoes in Hodh El Gharbi.

Mosquito genus	Mosquito species	Tintane (palm grove in the city)		
		08.09.2018		
		no.	(UG/BF)	%
*Culex*	*decens*	**8**	(3/5)	**4.60**
	*duttoni*	**8**	(8/0)	**4.60**
	*poicilipes*	**3**	(3/0)	**1.72**
	*quinquefasciatus*	**47**	(28/19)	**27.01**
	*univittatus*	**5**	(5/0)	**2.87**
	NI (f)	**4**	(4/0)	**2.30**
	NI (m)	**67**	(67/-)	**38.51**
*Aedes*	*vexans*	**9**	(0/9)	**5.17**
	NI (m)	**1**	(1/-)	**0.57**
*Anopheles*	*pharoensis*	**1**	(1/0)	**0.57**
	*gambiae*	**11**	(8/3)	**6.32**
	NI (f)	**1**	(1/0)	**0.57**
	NI (m)	**9**	(9/-)	**5.17**
Total		**174**	(138/36)	**100.00**

NI = (species) not identified

(m) = male mosquitoes

(f) = female mosquitoes

UG = unengorged mosquitoes (without visible blood meal)

BF = blood-fed mosquitoes

**Table 4:** Number of collected mosquitoes in Trarza.

Mosquito genus	Mosquito species	Rosso (city)		Rosso (cattle farm)^○^		Keurmacen (rice fields)		Total		
		08.12.2018		08.12.2018		08.12.2018				
		no.	(UG/BF)	no.	(UG/BF)	no.	(UG/BF)	no.	(UG/BF)	%
*Culex*	*antennatus*	**0**	(0/0)	**0**	(0/0)	**2**	(2(0)	**2**	(2(0)	**0.11**
	*decens*	**0**	(0/0)	**0**	(0/0)	**3**	(3/0)	**3**	(3/0)	**0.16**
	*neavei*	**0**	(0/0)	**2**	(2/0)	**4**	(4/0)	**6**	(6/0)	**0.33**
	*poicilipes*	**1**	(1/0)	**10**	(10/0)	**559**	(558/1)	**570**	(569/1)	**31.25**
	*quinquefasciatus*	**326** ^◆^	(310^◆^/16)	**47**	(47/0)	**85**	(85/0)	**458**	(442/16)	**25.11**
	*tritaeniorhynchus*	**3**	(3/0)	**21**	(19/2)	**44**	(43/1)	**68**	(65/3)	**3.73**
	*univittatus*	**0**	(0/0)	**0**	(0/0)	**6**	(6/0)	**6**	(6/0)	**0.33**
*Aedes*	*vexans*	**0**	(0/0)	**0**	(0/0)	**2**	(0/2)	**2**	(0/2)	**0.11**
	*aegypti*	**11** ^◆^	(8^◆^/3)	**0**	(0/0)	**0**	(0/0)	**11**	(8/3)	**0.60**
	NI (f)	**0**	(0/0)	**0**	(0/0)	**5**	(5/0)	**5**	(5/0)	**0.27**
*Anopheles*	*pharoensis*	**4**	(0/4)	**514** ^**+**^	(459^**+**^/55)	**12**	(12/0)	**530**	(471/59)	**29.06**
	*ziemanni*	**0**	(0/0)	**0**	(0/0)	**11**	(11/0)	**11**	(11/0)	**0.60**
	NI (f)	**1**	(0/1)	**0**	(0/0)	**2**	(2/0)	**3**	(2/1)	**0.16**
*Mansonia*	*uniformis*	**0**	(0/0)	**115**	(114/1)	**34**	(32/2)	**149**	(146/3)	**8.17**
Total		**346**	(322/24)	**709**	(651/58)	**769**	(763/6)	**1824**	(1736/88)	**100.00**

NI = (species) not identified

(m) = male mosquitoes

(f) = female mosquitoes

UG = unengorged mosquitoes (without visible blood meal)

BF = blood-fed mosquitoes

^**○**^ = RVF epizootic

^**+**^ = one pool tested positive for RVFV-specific RNA

^**◆**^ = one pool tested positive for dengue virus-specific RNA

#### III.I.II. RVFV detection

RVFV-specific RNA was found (1,783 copies/μl of viral RNA; Ct-value of 28) in one of the 779 mosquito samples tested. The sample contained a mosquito pool of ten female *Anopheles pharoensis* mosquitoes collected in December 2018 on the farm in Rosso (**Fig 1** and **Table 4**), where clinical RVF cases were observed in cattle.

The HTS results are shown in **Table 5**. Sequence alignments were generated for the eight contigs using all the available RVFV sequence data in GenBank (NCBI, Bethesda, USA). The contigs covered all three segments (S, M, L) and revealed a close relation to RVFV-isolates from South Africa, Mauritania and Namibia (accession numbers: OK392020, OK415590, OK415591, OK415592, OK415593, OK415594, OK415595, OK415596).

**Table 5:** Summary of HTS and alignment results of the RVFV-positive mosquito pool.

	Contig 1	Contig 2	Contig 3	Contig 4	Contig 5	Contig 6	Contig 7	Contig 8
**Accession number**	OK392020	OK415590	OK415591	OK415592	OK415593	OK415594	OK415595	OK415596
**Length (bp)**	821	772	1940	4417	461	200	2065	233
**Number of reads**	742	5719	1397	7188	4	2	934	1
**Genome segment**	S	S	L	L	M	M	M	M
**Most related sequence**	KX944815.1	EU312124	KX944862.1	KX944857.1	KY366332.1	KY366332.1	KX944836.1	KX944839.1
Identity (%, partial cds)	98.91	98.97	99.28	99.25	99.13	100	99.18	97.84
Country	South Africa	Namibia	South Africa	South Africa	Mauritania	Mauritania	South Africa	South Africa
Source (Host)	Bovine	Human	Ovine	Ovine	Human	Human	Bovine	Ovine
Collection date	2010	2004	2010	2009	2015	2015	2009	2010

In the attempt of virus isolation, no CPE was observed in either cell line during the first and second cell passage. RVFV qRT-PCR revealed the presence of 7.54 copies/μl viral RNA on Vero 76 cells and 3.22 copies/μl viral RNA on C6/36 cells in cell culture supernatants of the first cell passage after seven days incubation. However, cell culture supernatants of the second cell passage showed no evidence of RVFV-specific RNA. Likewise, in IIFA no evidence of replicable RVFV was observed in these cells, indicating that viral RNA detected in supernatants of the first cell passage was derived from the original sample.

#### III.I.III. *Flavivirus* and *Orthobunyavirus* detection

In qRT-PCR, two out of 779 mosquito samples showed melting curves similar to that of Usutu virus (positive control), thus indicative for flaviviruses. Sequencing of qRT-PCR-products of these samples revealed the presence of dengue virus (DENV, family *Flaviviridae*, genus *Flavivirus*) RNA in both mosquito pools. The first sample consisted of a pool of eight female *Aedes aegypti* mosquitoes that were captured in the town of Rosso (**Fig 1**) in December 2018. The second sample contained a pool of ten female *Culex quinquefasciatus* mosquitoes that were captured at the same collection site (**Table 4**).

**Fig 2:**
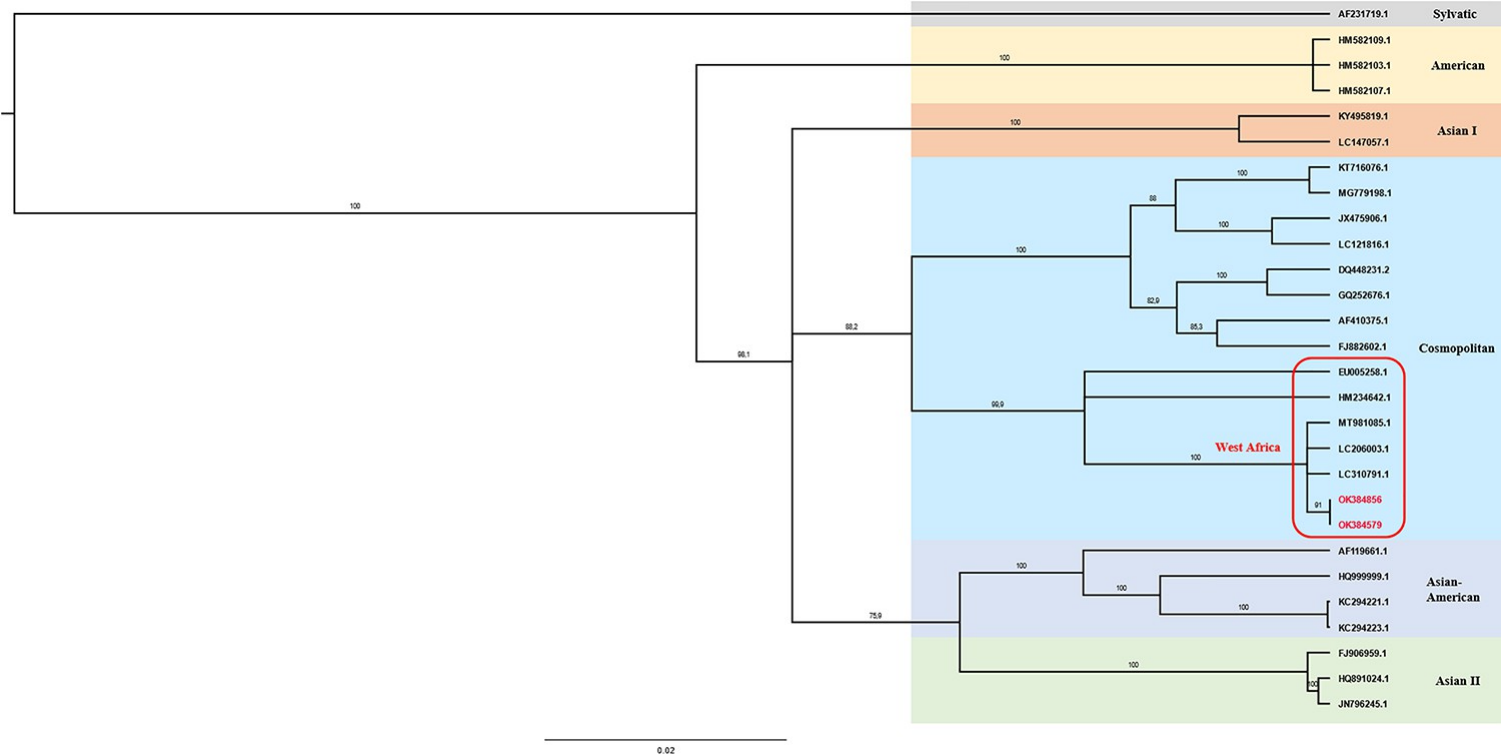
Phylogenetic tree of DENV-2 genotypes. Dengue virus partial sequences were analyzed by MEGA software with bootstrap support of 1000 replicates. The recent sequences are presented in red.

Using HTS, partial genome sequences of both samples could be generated. For the positive *Aedes* pool a sequence of 572 bp (accession number: OK384856) was detected (530 reads). A sequence of 5076 bp (5965 reads) could be generated for the positive *Culex* sample (accession number: OK384579). The alignment of these sequences revealed highest identity with other DENV strains isolated in 2018 from human sera in West Africa. The *Aedes aegypti*-derived DENV sequence had 99.8% sequence homology with an October 2018 isolate from Mauritania (MT981085.1) and the *Culex quinquefasciatus*-derived sequence had 99.9% sequence homology with a December 2018 isolate from Senegal (MT981011.1). Both partial sequences are belonging to the DENV-2 serotype (**S2 Fig**) and cluster into the Cosmopolitan genotype, seeming phylogenetically close to other West African strains (**Fig 2**).

In the attempt of virus isolation of these two samples that showed evidence of dengue virus-derived RNA, neither CPE nor dengue virus RNA was detected in either cell passage.

For orthobunyaviruses, no evidence of viral RNA was found in the mosquito samples.

#### III.I.IV. Blood meal analysis

**Fig 3:**
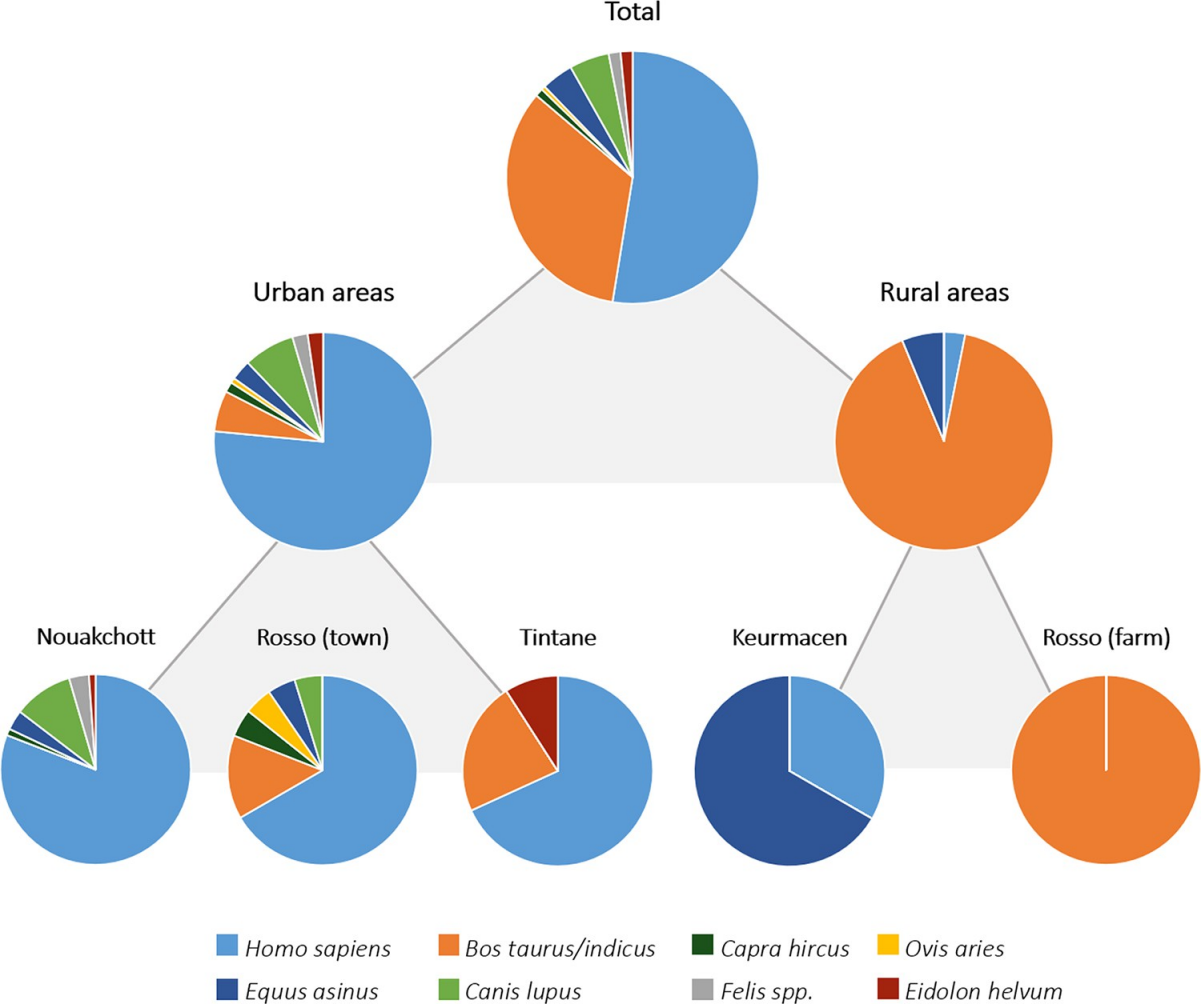
Allocation of blood meal sources. Results of the different sampling sites in the city of Nouakchott are displayed together.

The source of blood meals was identified for a total of 196 out of the 258 blood-fed mosquitoes. The cytochrome b and 16S ribosomal RNA banding patterns and sequences yielded identical results for 160 blood meal samples. For 22 blood meal samples, only specific cytochrome b banding patterns and sequences were found, while for 14 other blood meal samples, only specific 16S ribosomal RNA banding patterns and sequences were detected (**S1 Table**). Engorged mosquitoes of which the identification of blood meal was successful were among eleven of the captured species (**Table 6**). Over 50% of the mosquitoes fed on humans (*Homo sapiens*), followed by almost 35% of the mosquitoes that fed on cattle (*Bos taurus/indicus*) (**Fig 3**). For about 15% of the mosquitoes, other feeding hosts were detected, including small ruminants (*Capra hircus*, *Ovis aries*), donkeys (*Equus asinus*), cats (*Felis* spp.) and dogs (*Canis lupus*; dog-like carnivores) as well as straw-colored fruit bats (*Eidolon helvum*) (**Table 6**). Most mosquitoes collected in urban areas fed on humans whereas those captured in rural areas mostly fed on cattle (captured next to a cattle farm) and donkeys (near rice fields) (**Fig 3**).

**Table 6:** Blood meal sources (hosts) of blood-fed mosquitoes.

Mosquito		Hosts								
Genus	Species	*Homo sapiens*	*Bos taurus / indicus*	*Capra hircus*	*Ovis aries*	*Equus asinus*	*Canis lupus* *	*Felis spp*.	*Eidolon helvum*	Total
*Culex*	*antennatus*	3	0	0	0	0	0	0	0	3
	*decens*	3	0	0	0	0	0	0	0	3
	*poicilipes*	0	0	0	0	1	0	0	0	1
	*quinquefasciatus*	67	1	2	0	2	8	1	3	84
	*tritaeniorhynchus*	2	2	0	0	1	0	0	0	5
	*univittatus*	19	0	0	0	1	1	2	0	23
*Aedes*	*vexans*	4	2	0	0	2	1	0	0	9
	*aegypti*	2	0	0	0	0	0	0	0	2
*Anopheles*	*pharoensis*	1	57	0	1	0	0	0	0	59
	*gambiae*	1	2	0	0	0	0	0	0	3
	NI	0	1	0	0	0	0	0	0	1
*Mansonia*	*uniformis*	1	1	0	0	1	0	0	0	3
Total		103	66	2	1	8	10	3	3	196

NI = (species) not identified

* = including subspecies of *Canis lupus* such as *C*.*l*. *familiaris*

### III.II. Serum samples

#### III.II.I. RVFV detection

**Fig 4:**
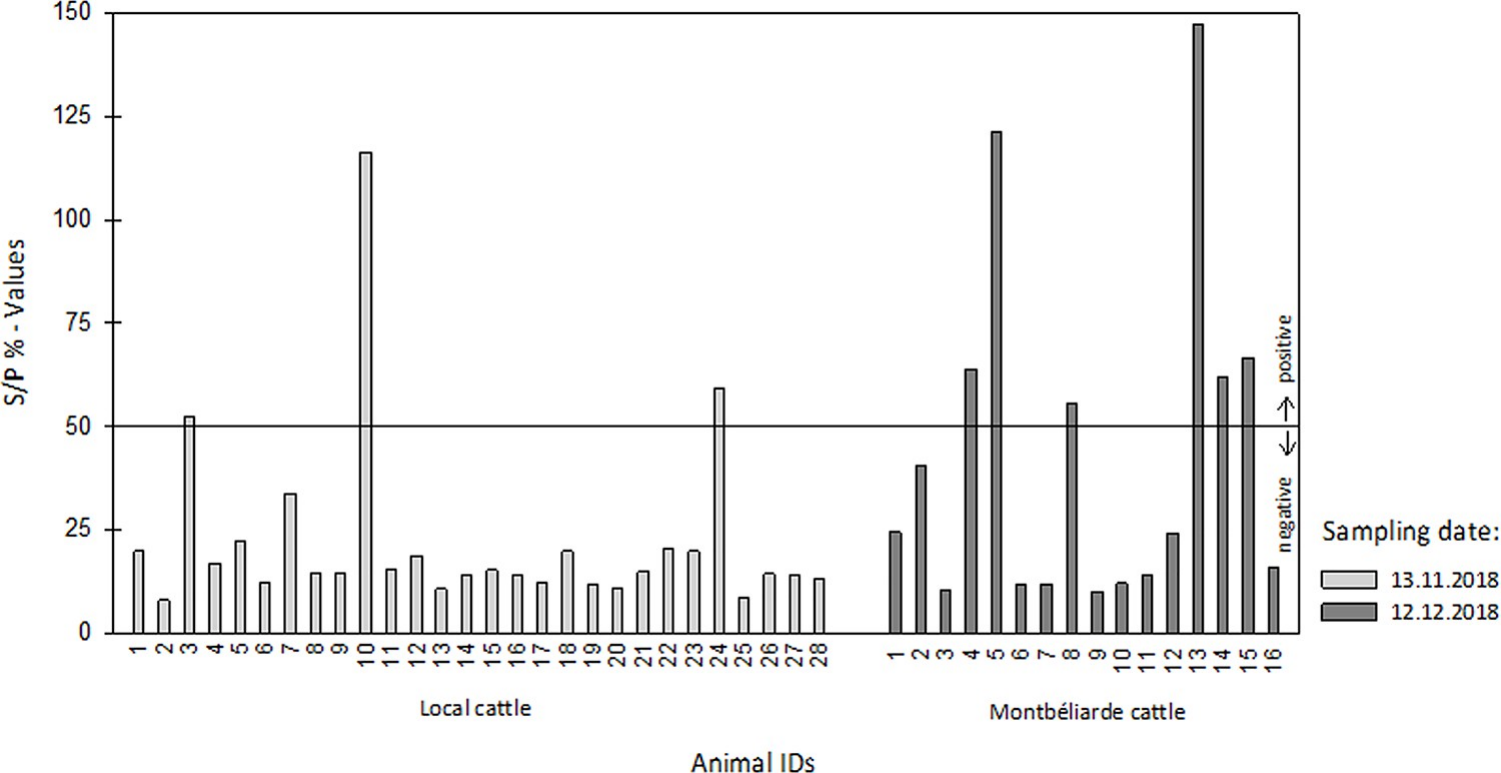
IgM Capture ELISA. S/P % = corrected optic density_sample_ / corrected optic density_positive control_ x 100.

In December, when RVF disease (abortions, fever, hypersalivation) was observed on the farm in Rosso, six out of 16 (37.5%) Montbéliarde cattle had detectable IgM antibodies against RVFV. In sera of local cattle sampled one month earlier on the same farm, IgM antibodies against RVFV were detected in three out of 28 (10.7%) animals (**Fig 4**). No evidence of RVFV-derived RNA was detected in any of the 44 tested sera and the examination of IgM positive sera revealed no evidence of replicable RVFV.

#### III.II.II. *Flavivirus* and *Orthobunyavirus* detection

No evidence of *Flavivirus*-specific or *Orthobunyavirus*-specific RNA was found in any of the 44 serum samples.

## IV. Discussion

One of the aims of this study was to elucidate the abundance of RVFV-transmitting mosquitoes and the presence of the virus in vectors in Mauritania. As amplification hosts that promote RVFV maintenance during the enzootic cycle remain unidentified to date [5], blood meals of mosquitoes were analyzed to obtain information on mosquito host preferences. Furthermore, the study aimed to examine the occurrence of flavi- and orthobunyaviruses in mosquitoes in Mauritania.

To achieve these objectives, 4,950 mosquitoes were collected during 2018 in three southern regions of Mauritania (**Fig 1**) where RVF outbreaks have previously occurred [9,40]. Fourteen different mosquito species were identified, comprising four genera that have already been described in Mauritania (**Table 1**) [12]. Consistent with previous studies, *Culex* spp. constituted the most abundant mosquito genus [13,41]. As species abundance of mosquitoes in Mauritania varies depending on the season [15] and most locations were only sampled once during the year, limited conclusions can be drawn about the seasonal species distribution in the regions. Furthermore, it is possible that diurnally active species, such as many *Aedes* spp., may have been underrepresented due to the collection method used.

Eleven of the captured mosquito species have previously been described as RVFV carriers (**Table 1**) [8]. In this study, we found evidence of RVFV in a pool of female *Anopheles pharoensis* mosquitoes (**Table 4**). RVFV infection of this species has previously been detected in Kenya [7,8], but to our knowledge this is the first description of RVFV in *An*. *pharoensis* in Western Africa. This finding emphasizes the need of performing vector competence studies, as only these can clarify whether the mosquito species is capable of transmitting RVFV from one host to another. Comparison of the RVFV contigs using the BLAST tool (NCBI, Bethesda, USA) revealed a close relationship to isolates from South Africa, Namibia, and Mauritania (**Table 5**), but it is difficult to draw conclusions about a geographic phylogeny from these results due to the overall highly conserved nature of the RVFV genome [42]. The RVFV-positive mosquitoes were collected on a farm in Rosso (**Fig 1**) while cattle on the farm exhibited symptoms of RVF disease. The recency of the RVF epizootic was confirmed by the detection of RVFV-specific IgM antibodies in bovine sera collected on the farm, as IgM antibodies in cattle persist only during the first few months after RVFV infection [2]. However, it can be assumed that the epizootic had already started prior to the observed clinical manifestation, since IgM antibodies were also detected in sera taken a month earlier and no evidence of the virus itself was found in any of the blood samples. Viremia in infected cattle lasts only for about seven days [43] and had probably already subsided in the sampled animals.

In order to investigate RVFV maintenance, the majority of mosquitoes (4,241 individuals) in this study were collected during the absence of reported RVF cases. Other studies have previously revealed the presence of the virus in mosquitoes during the enzootic cycle [44,45], but these data remain limited because virus detection during these periods is difficult due to low mosquito infection rates [46,47] and sparse transmission to susceptible hosts [16]. However, evidence of active RVFV circulation was found only in those mosquitoes that were additionally captured (709 individuals) in close proximity to animals with confirmed RVF disease. In addition, transovarial RVFV transmission within *Aedes* spp. mosquitoes is believed to be a key factor in RVFV maintenance [1,48]. Yet there is little data available to support this type of maintenance [49], and it has never been confirmed in Western Africa [1]. To verify its occurrence within different mosquito genera in Mauritania, in addition to female mosquitoes, captured males were also examined, which do not feed on vertebrates but exclusively on plants. However, none of the 571 tested male mosquitoes caught in the absence of RVF cases showed evidence of RVFV infection. Future collection and analyses of male mosquitoes, as well as of larval and egg stages of mosquitoes during epidemics will help to clarify the role of vertical RVFV transmission in Mauritania.

Wildlife capable of acting as amplification hosts may be another factor favoring RVFV maintenance [1]. To identify mosquito hosts and thus animals possibly involved in the RVFV transmission cycle, blood meals of 258 engorged mosquitoes were examined. Similar to other studies in Mauritania, where mosquitoes of the genus *Anopheles* spp. were analyzed, the vast majority of mosquitoes fed on humans (urban areas) and cattle (rural areas) (**Fig 3**) [50,51], depending on the availability of these feeding hosts. This reflects the potential risk posed by infected mosquitoes, as both humans and cattle are known to be highly susceptible to RVFV [4]. A small proportion of the engorged mosquitoes used other hosts to feed. In line with previous investigations, mosquitoes also fed on small ruminants (goats, sheep), donkeys and dogs (dog-like carnivores) [51,52], and this study additionally identified cats and straw-colored fruit bats (*Eidolon helvum*) as sources of blood meals (**Table 6**). The identification of fruit bat blood in the mosquitoes is of particular interest as bats have been repeatedly suggested as RVFV reservoir [5,53,54]. The host-vector interaction between *Eidolon helvum* and mosquitoes has also been reported in other regions with endemic RVFV circulation [55]. Moreover, RVFV and specific antibodies have been detected in several bat species in Africa [53,56,57,58], and experimental infection studies have demonstrated a variety of bat species to be susceptible to the virus [5,53,59]. However, the definite role of bats in RVFV ecology remains undetermined [5] and should be investigated more closely. Interestingly, no birds were found as feeding hosts in this study, which is in contrast to studies in North America that found birds to be the primary hosts of *Culex* mosquitoes [60].

Furthermore, evidence of DENV circulation was found in two mosquito pools collected in December in the town of Rosso (**Fig 1** and **Table 4**). To our knowledge, this is the first description of DENV detected in mosquitoes in Mauritania. Dengue fever is caused by four DENV serotypes (DENV 1–4). An additional serotype (DENV-5) has been described, but it is currently limited to a single outbreak in Asia [61]. The DENV strains detected in this study showed highest identity with DENV-2 strains isolated in West Africa (**Fig 2**). This is not surprising given the results of other studies that have described DENV-2 as the dominant serotype in Africa [62]. The closest genetic proximity was found to two sequences isolated from human sera in Mauritania and in Senegal, also in 2018. As the DENV-positive mosquitoes in this study were collected in Rosso, which is close to the Senegalese border, these results, alongside with the previously detected sequences in patients, prove the epidemic circulation of DENV-2 in this geographic region. The first evidence of DENV-2 was found in a mosquito pool containing female *Aedes aegypti* mosquitoes, primary vectors of the virus [23]. The second DENV-2-positive pool consisted of female *Culex quinquefasciatus* mosquitoes known as vectors for West Nile virus, another virus of the genus *Flavivirus* [63,64]. Early on, this species has been discussed as potential vector for DENV, but published data are very contradictory [23]. However, the virus has been previously isolated from other *Culex* spp. mosquitoes [65], indicating the need for further studies to evaluate the role of these mosquitoes in DENV transmission.

To conclude, evidence of RVFV as wells as DENV was found in mosquitoes collected in Mauritania in 2018. Furthermore, it was demonstrated that mosquitoes feed on fruit bats, which are suspected to be involved in RVFV maintenance. In order to gain a thorough understanding of RVFV ecology, further in-depth studies are required to investigate factors promoting RVFV maintenance. As discussed by *Bhatt* et al., the burden caused by DENV in Africa may be largely underestimated [20]. Its occurrence in Mauritania needs to be investigated further, since very little is known to date. Although no other mosquito-borne viruses were detected in this study, several other arboviruses are circulating in Mauritania, threatening human and animal health [15,17,18]. This again underlines the need for research on the occurrence of arboviruses and their vectors in order to be able to promote their control and prevention.

## Supporting information

S1 Table Comparison of blood meal source results obtained by the different PCRs. **A)** Cytochrome b PCR. **B)** 16S ribosomal RNA PCR.
(DOCX)

S1 Fig Numbers of captured mosquitoes over the year 2018. (TIF)

S2 Fig Phylogenetic tree of DENV serotypes (DENV 1–4). The recent sequences are presented in red.
(TIF)
