# Supplementary material for: Mosquito survey in Mauritania: Detection of Rift Valley fever virus and dengue virus and the determination of feeding patterns
Source: PLoS Negl Trop Dis. 2022 Apr 15;16(4):e0010203. doi: 10.1371/journal.pntd.0010203 (PMC9113561; doi:10.1371/journal.pntd.0010203)
Supplement: S1 Table — A) Cytochrome b PCR. B) 16S ribosomal RNA PCR. (DOCX) [file pntd.0010203.s001.docx]

**S1 Table.** Comparison of blood meal source results obtained by the different PCRs.

**A)** Cytochrome b PCR

| Mosquito | | Hosts | | | | | | | |  |
| --- | --- | --- | --- | --- | --- | --- | --- | --- | --- | --- |
| Genus | Species | ***Homo sapiens*** | ***Bos taurus / indicus*** | ***Capra hircus*** | ***Ovis aries*** | ***Equus asinus*** | ***Canis lupus**** | ***Felis spp.*** | ***Eidolon helvum*** | Total |
| *Culex* | *antennatus* | 3 | 0 | 0 | 0 | 0 | 0 | 0 | 0 | 3 |
|  | *decens* | 3 | 0 | 0 | 0 | 0 | 0 | 0 | 0 | 3 |
|  | *poicilipes* | 0 | 0 | 0 | 0 | 1 | 0 | 0 | 0 | 1 |
|  | *quinquefasciatus* | 61 | 1 | 2 | 0 | 0 | 4 | 1 | 3 | 72 |
|  | *tritaeniorhynchus* | 2 | 2 | 0 | 0 | 1 | 0 | 0 | 0 | 5 |
|  | *univittatus* | 19 | 0 | 0 | 0 | 0 | 1 | 2 | 0 | 22 |
| *Aedes* | *vexans* | 4 | 2 | 0 | 0 | 2 | 1 | 0 | 0 | 9 |
|  | *aegypti* | 2 | 0 | 0 | 0 | 0 | 0 | 0 | 0 | 2 |
| *Anopheles* | *pharoensis* | 0 | 57 | 0 | 1 | 0 | 0 | 0 | 0 | 58 |
|  | *gambiae* | 1 | 2 | 0 | 0 | 0 | 0 | 0 | 0 | 3 |
|  | NI | 0 | 1 | 0 | 0 | 0 | 0 | 0 | 0 | 1 |
| *Mansonia* | *uniformis* | 1 | 1 | 0 | 0 | 1 | 0 | 0 | 0 | 3 |
| Total | | 96 | 66 | 2 | 1 | 5 | 6 | 3 | 3 | 182 |
| **B)** 16S ribosomal RNA PCR | | |  |  |  |  |  |  |  |  |
| Mosquito | | Hosts | | | | | | | |  |
| Genus | Species | ***Homo sapiens*** | ***Bos taurus / indicus*** | ***Capra hircus*** | ***Ovis aries*** | ***Equus asinus*** | ***Canis lupus**** | ***Felis spp.*** | ***Eidolon helvum*** | Total |
| *Culex* | *antennatus* | 3 | 0 | 0 | 0 | 0 | 0 | 0 | 0 | 3 |
|  | *decens* | 2 | 0 | 0 | 0 | 0 | 0 | 0 | 0 | 2 |
|  | *poicilipes* | 0 | 0 | 0 | 0 | 1 | 0 | 0 | 0 | 1 |
|  | *quinquefasciatus* | 53 | 1 | 2 | 0 | 2 | 8 | 1 | 3 | 70 |
|  | *tritaeniorhynchus* | 1 | 2 | 0 | 0 | 1 | 0 | 0 | 0 | 4 |
|  | *univittatus* | 15 | 0 | 0 | 0 | 1 | 1 | 2 | 0 | 19 |
| *Aedes* | *vexans* | 3 | 2 | 0 | 0 | 2 | 1 | 0 | 0 | 8 |
|  | *aegypti* | 2 | 0 | 0 | 0 | 0 | 0 | 0 | 0 | 2 |
| *Anopheles* | *pharoensis* | 1 | 57 | 0 | 1 | 0 | 0 | 0 | 0 | 59 |
|  | *gambiae* | 1 | 1 | 0 | 0 | 0 | 0 | 0 | 0 | 2 |
|  | NI | 0 | 1 | 0 | 0 | 0 | 0 | 0 | 0 | 1 |
| *Mansonia* | *uniformis* | 1 | 1 | 0 | 0 | 1 | 0 | 0 | 0 | 3 |
| Total | | 82 | 65 | 2 | 1 | 8 | 10 | 3 | 3 | 174 |

NI = (species) not identified

* = including subspecies of *Canis lupus* such as *C. l. familiaris*
