# Supplementary figures and images for: Mosquito survey in Mauritania: Detection of Rift Valley fever virus and dengue virus and the determination of feeding patterns
Source: PLoS Negl Trop Dis. 2022 Apr 15;16(4):e0010203. doi: 10.1371/journal.pntd.0010203 (PMC9113561; doi:10.1371/journal.pntd.0010203)

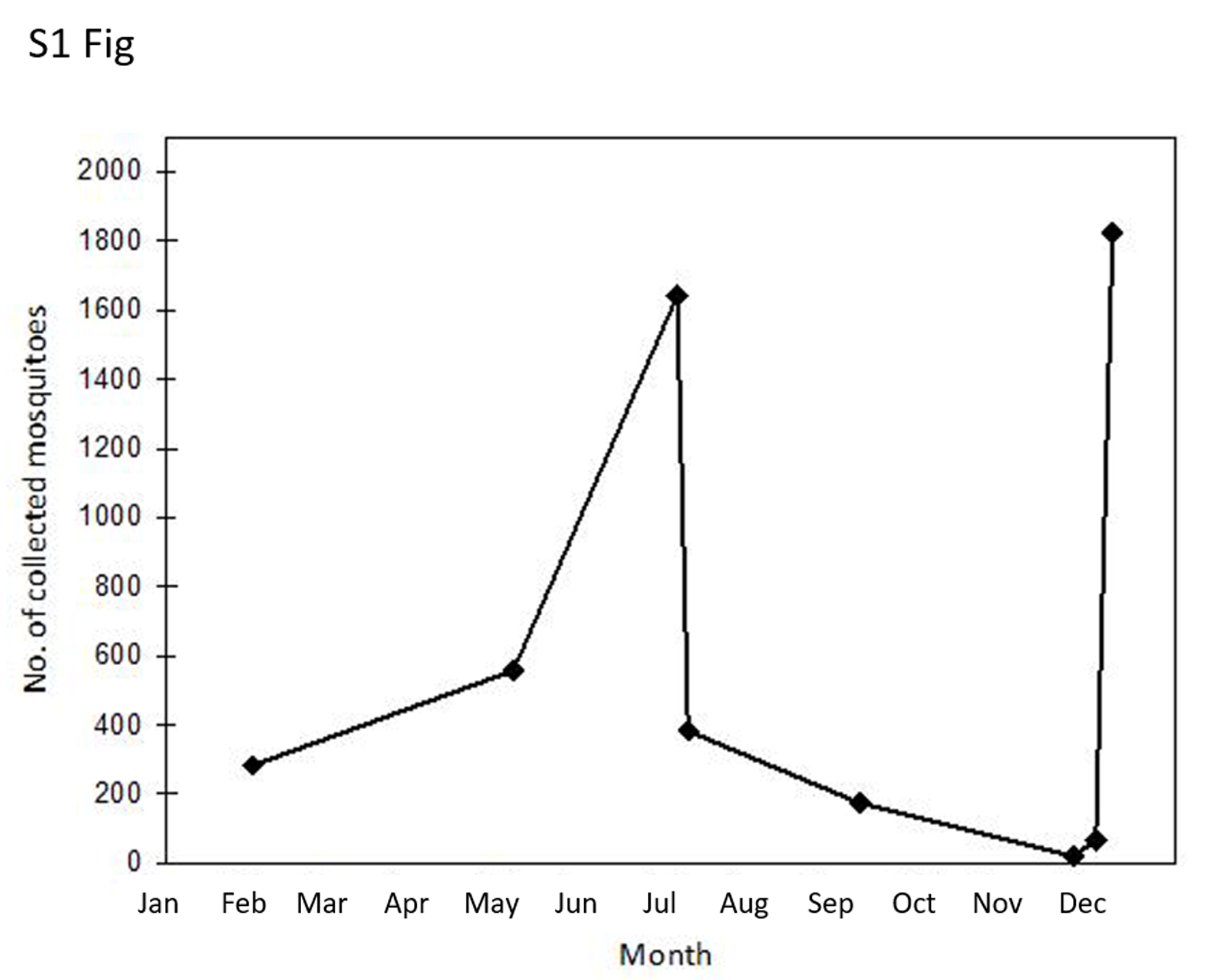

Supplement: S1 Fig — (TIF) [file pntd.0010203.s002.tif]

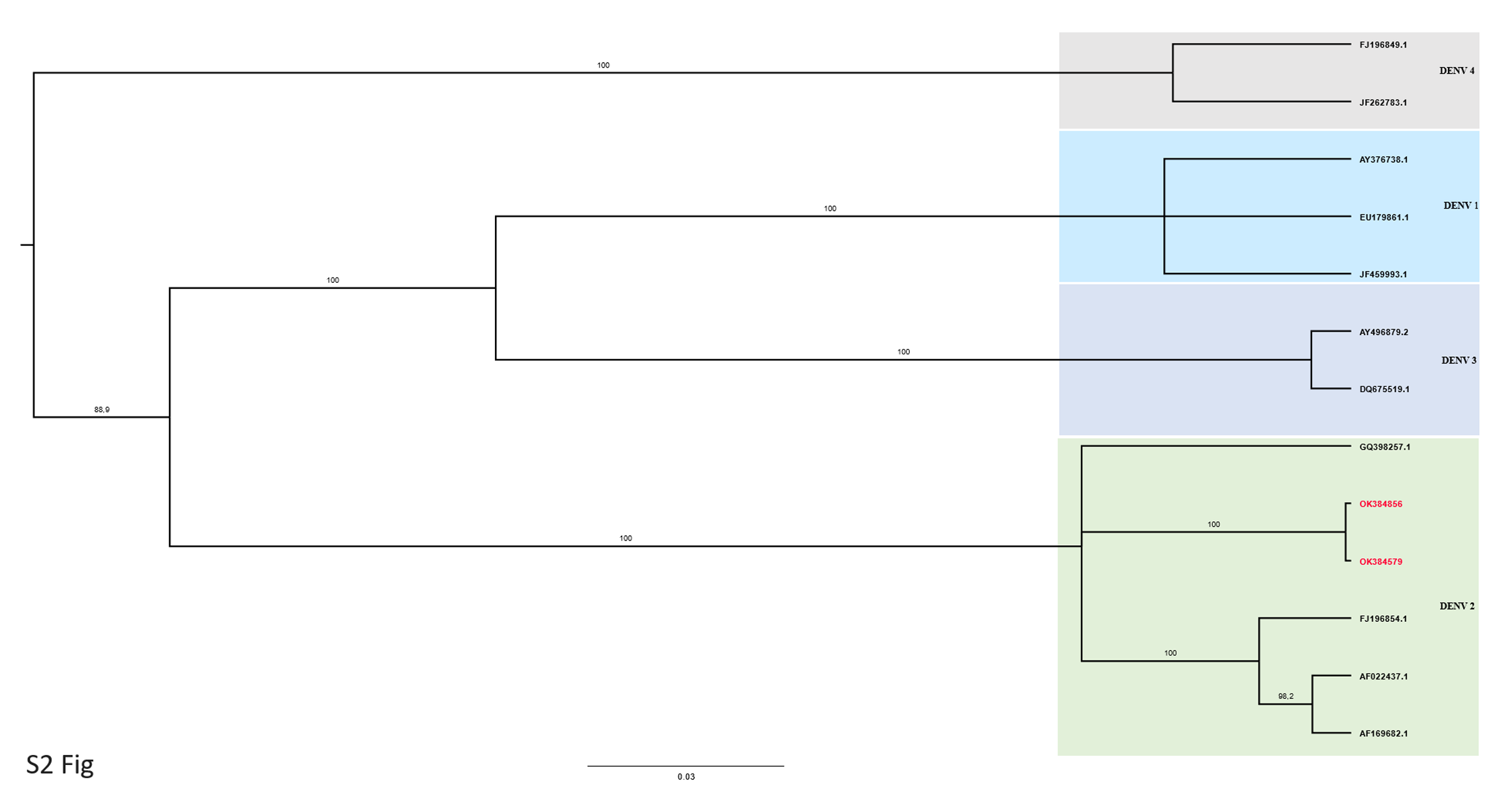

Supplement: S2 Fig — The recent sequences are presented in red. (TIF) [file pntd.0010203.s003.tif]
